# App-Based Digital Therapeutics Integrating Continuous Glucose Monitoring for Glycemic Control in Type 2 Diabetes: Prospective Observational Cohort Study

**DOI:** 10.2196/86651

**Published:** 2026-07-08

**Authors:** Banshi Saboo, Mudit Sabharwal, Mihir Gharia, Devina Aswal, Parth Modi, Twinkle Maheshwari, Jaymin Parikh, Simpel Saanyal, Jaikishan Agrawal

**Affiliations:** 1 Diabetes Care and Hormone Clinic, Dia Care Research, Ahmedabad, Gujarat, India; 2 Department of Diabetes and Endocrinology, Dharma Diabetes and Metabolic Clinics, Delhi, India; 3 Department of Medical Affairs, Tatvacare, Purple H 202, Maple County 2, Sindhu Bhavan Road Thaltej, Ahmedabad, Gujarat, 380059, India, +91 9769991897

**Keywords:** type 2 diabetes mellitus, continuous glucose monitoring, digital therapeutics, lifestyle intervention, cognitive behavioral therapy

## Abstract

**Background:**

Digital therapeutics integrating continuous glucose monitoring (CGM) with personalized lifestyle coaching can enhance glycemic control in individuals with type 2 diabetes mellitus (T2DM). However, real-world evidence evaluating such multicomponent interventions—combining CGM with nutrition coaching, physiotherapy, and cognitive behavioral therapy within a unified digital platform—remains limited in South Asian populations.

**Objective:**

This study aimed to evaluate the effectiveness of an app-based lifestyle-integrated intervention program (Glycemic Lifestyle Intervention in Diabetes Empowerment–CGM; GLIDE-C) on glycemic, anthropometric, lipid profile, quality of life (QoL), and behavioral outcomes, as well as outcomes related to HEOR (health economics and outcomes research), in adults with mildly uncontrolled T2DM.

**Methods:**

A prospective observational cohort study was conducted among adults aged ≥18 years with confirmed T2DM and baseline hemoglobin A_1c_ (HbA_1c_) between 7.5% and 9.5%. The intervention was delivered via the Goodflip mobile app, including a 15-day CGM period and a structured analysis of glucose variations, among other clinical assessments, with follow-up on days 15, 30, and 60. The digital intervention combined CGM-guided personalized diet, exercise, and cognitive behavioral therapy plans delivered through the mobile app, supported by multidisciplinary coaching. Primary outcomes were changes in HbA_1c_, fasting plasma glucose, postprandial glucose, and CGM metrics; secondary outcomes included lipid profile, anthropometric indices, QoL, and behavioral determinants of glycemic improvement.

**Results:**

Eighteen participants (mean age 49.45, SD 10.91 years; n=11, 61.1% male) completed the program. Mean HbA_1c_ decreased by 0.59% (*P*=.05), with postprandial glucose decreasing by 25.42 mg/dL (*P*=.06) and time in range increasing by 9.98%. Modest reductions, consistent with the short intervention duration, occurred in low-density lipoprotein cholesterol (−8.38 mg/dL), body fat (−1.13 kg), and waist circumference (−1.56 cm). QoL improved significantly for sleep quality (*P*=.04). Improvements were also observed for motivation (*P*=.06), stress management (*P*=.08), and mood (*P*=.12), although these changes were not statistically significant. Behavioral analysis demonstrated strong associations with glycemic outcomes; all participants who reduced their consumption of energy-dense, nutrient-poor foods experienced improvements in HbA_1c_, whereas those who did not reduce energy-dense, nutrient-poor intake showed less favorable outcomes.

**Conclusions:**

The novel GLIDE-C program demonstrated clinically meaningful improvements in glycemic control, behavioral outcomes, and QoL in adults with T2DM, without pharmacological modification. These findings strengthen the role of integrated, CGM-guided digital therapeutic platforms as an effective adjunct to the conventional standard of care.

## Introduction

Type 2 diabetes mellitus (T2DM) constitutes one of the most consequential noncommunicable disease epidemics of the 21st century. The International Diabetes Federation Diabetes Atlas (11th edition) estimates that 589 million adults worldwide were living with diabetes in 2024, a figure projected to reach 853 million by 2050, with the largest relative increases expected in low- and middle-income countries [1]. The landmark national Indian Council of Medical Research-India Diabetes cross-sectional study reported a diabetes prevalence of 11.4% among Indian adults, with an estimated 101 million people affected and a further 136 million living with prediabetes across the country [2]. South Asian individuals are phenotypically predisposed to insulin resistance at lower BMIs, develop T2DM at a younger age, and carry a higher burden of complications relative to their Western counterparts [3]. Effective glycemic management is therefore of paramount clinical importance. Glycemic monitoring relies on hemoglobin A_1c_ (HbA_1c_) and self-monitoring of blood glucose (SMBG). While HbA_1c_ remains the principal marker of long-term glycemic control, it is insensitive to intraday glucose variability, postprandial excursions, and nocturnal hypoglycemia. Similarly, SMBG provides only episodic data and is highly dependent on patient adherence and technique [4]. Continuous glucose monitoring (CGM), a more advanced glucose monitoring technology, enables real-time assessment of interstitial glucose levels and provides a more comprehensive view of glycemic patterns and variability [4]. However, translating these insights to a sustained behavioral change requires a structured clinical, nutritional, and psychological support framework.

A 2024 systematic review and meta-analysis demonstrated that real-time CGM and intermittently scanned CGM both produce clinically meaningful HbA_1c_ reductions compared with SMBG across insulin and noninsulin treatment regimens [5]. Similar results have been demonstrated in larger cohorts (n=74,679), with reductions in all-cause hospitalizations and acute diabetes-related emergencies [6]. However, certain limitations persist, such as the results possibly being attenuated without behavioral and lifestyle components [7]. The concurrent evidence base for digital therapeutics and app-based interventions in T2DM is characterized by heterogeneity in both design and outcomes. Most interventions focus on behavioral therapy, nutrition guidance, physical activity, or various combinations thereof and are typically not comprehensive [7-9]. In the Indian context, real-world digital therapeutics programs have demonstrated improvements in glycemic and weight-related outcomes among people with T2DM [10,11]. A diabetes CGM digital therapeutics program, closely analogous to prior real-world evaluation, demonstrated a reduction in all glycemic parameters but lacked a structured incorporation of physiotherapy, cognitive behavioral therapy (CBT), or multidomain quality of life (QoL) assessment [10]. Collectively, these limitations, due to single-component or partially integrated interventions, can achieve improvements in isolation; however, behavior change sustainability and patient-centered outcomes remain inconsistently addressed [12,13].

Existing literature reveals critical gaps. There is a lack of integrated multidisciplinary digital therapeutics combining CGM-guided glycemia, personalized nutrition, exercise prescription, and CBT. Real-world evidence remains limited beyond controlled trials. South Asian or Indian populations are underrepresented despite their high T2DM burden [3,11]. No studies evaluate CGM metrics, HbA_1c_, anthropometrics, behavior, QoL, and health economics in a single program.

To address these gaps, this study introduces a novel, fully integrated digital therapeutics model, GLIDE-C, which uniquely combines CGM-guided glycemic monitoring with personalized nutrition, structured physiotherapy, and CBT within a single digital ecosystem. Unlike prior interventions that evaluate isolated components or limited outcomes, this study simultaneously assesses multidomain end points, including glycemic metrics such as HbA_1c_ and CGM parameters, anthropometrics, behavioral changes, QoL, and exploratory health economic outcomes in a real-world Indian population. The aim of this study was to evaluate the real-world effectiveness of the GLIDE-C program over 90 days among Indian adults with T2DM.

## Methods

### Study Design and Setting

This prospective, observational cohort study was conducted at DiaCare Research, Ahmedabad, between June 2024 and December 2024 using deidentified program data from adults enrolled in the GLIDE-C program, a structured 90-day digital therapeutics intervention delivered via the Goodflip mobile app. This observational study was supported by a multidisciplinary health coaching team.

### Participants

Eligible participants were adults aged ≥18 years with a confirmed diagnosis of T2DM and a baseline HbA_1c_ between 7.5% and 9.5%. All participants were receiving ongoing standard-of-care antidiabetic therapy as prescribed by their treating physician. Additional eligibility criteria included ownership of a smartphone with internet access and sufficient English literacy to engage with the program content and complete study questionnaires. Participants were excluded if they were currently or had recently been involved in other structured lifestyle intervention programs, were taking weight-loss supplements or pharmacotherapy, had no recent intensification or initiation of new antidiabetic medications in the month preceding enrollment, were pregnant or breastfeeding within the past 3 months, had undergone recent surgery, or had advanced chronic kidney disease (stage 4 or 5) or liver cirrhosis.

### GLIDE-C Program

GLIDE-C is a multicomponent integrated digital therapeutics program designed to support glycemic control through personalized lifestyle modification. The intervention was delivered through the Goodflip mobile app (version 5.2.23; iOS and Android), which integrates CGM data, Internet of Things (IoT) devices, educational content, behavioral therapy modules, and 2-way communication with health coaches. Upon enrollment, participants completed a baseline consultation where demographic characteristics, dietary habits, physical activity levels, and QoL measures were captured. QoL domains were assessed using custom, program-developed scales and not externally validated instruments. Following this assessment, each participant was assigned a dedicated multidisciplinary team comprising a dietitian, physiotherapist, and CBT.

**Figure 1.**
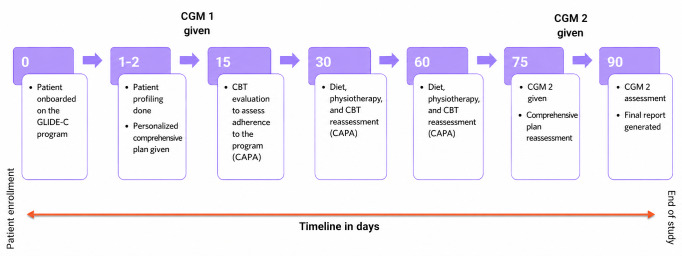
Participants’ journey through the 90-day GLIDE-C program. CAPA: corrective and preventive actions; CBT: cognitive behavioral therapy; CGM: continuous glucose monitoring.

The app visualized key CGM metrics, time in range (TIR), time above range (TAR), and time below range (TBR) and generated personalized glucose alerts adjustable to clinical thresholds. At the end of the initial CGM period, a structured root cause analysis (for diet, behavior, and exercise) was conducted, followed by modifications to the intervention plan as needed. The nutrition and physiotherapy coaches guided the patients with personalized plans, while the CBT interventions focused on addressing motivational barriers, stress management, and emotional eating using mindfulness training, relaxation techniques, and cognitive restructuring. Participants’ journey through the program is depicted in Figure 1.

### Outcomes

The primary outcomes were changes from baseline to day 90 in HbA_1c_, fasting plasma glucose, postprandial glucose, CGM-derived measures (TIR, TAR, and TBR), anthropometric parameters (including weight, BMI, waist circumference, and body fat percentage), and lipid profile measures. Secondary outcomes included changes in QoL parameters and exploratory health economics and outcomes research (HEOR) measured through reduction in insulin dose and extension in follow-up visits calibrated at the end of the study.

### Data Analysis

The deidentified data for all the participants were taken from the Goodflip mobile app. Continuous variables were summarized as mean (SD). Normality of distribution was assessed using the Shapiro-Wilk test. For paired baseline and postintervention comparisons, the paired *t* test (2-tailed) was applied to normally distributed variables, while the Wilcoxon signed-rank test was used for nonnormally distributed variables. Percentage changes from baseline were calculated for each outcome. Associations between behavioral changes (nutrient-rich food intake; energy-dense, nutrient-poor [EDNP] food intake; and weekly exercise) and HbA_1c_ improvement were explored descriptively. All analyses were performed using SPSS (version 27.0; IBM Corp), with 2-tailed *P* values <.05 considered statistically significant.

### Ethical Considerations

The study protocol was reviewed and approved by the institutional ethics committee of Aatman Hospital, Ahmedabad, India (EC/NEW/INST/2021/11802). The study was prospectively registered with the Clinical Trials Registry-India (CTRI/2024/08/071850). All study procedures adhered to the principles outlined in the Declaration of Helsinki and its subsequent amendments. Electronic informed consent was obtained from each participant before enrollment; participants were informed of the study purpose, the voluntary nature of participation, and their right to withdraw at any time without any change to their ongoing standard of care. The data collected were deidentified prior to analysis, with restricted access to authorized personnel only.

## Results

### Participant Characteristics

**Figure 2.**
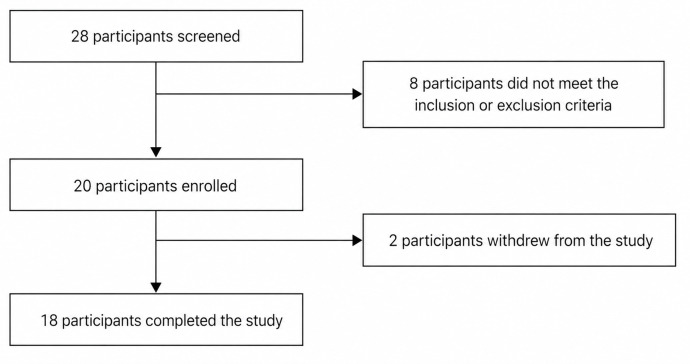
Participant flow.

A total of 20 participants were enrolled after screening 28 participants; 18 participants completed the 90-day program and were included in the final analysis (90% completion rate). The 2 noncompleters withdrew voluntarily for reasons unrelated to the intervention or adverse events; no protocol-mandated withdrawals occurred (Figure 2). Baseline anthropometric, glycemic, lipid, and CGM parameters are summarized in Table 1.

**Table 1.** Baseline demographic and clinical characteristics of participants (N=18).

Variable	Values
Age (years), mean (SD)	49.45 (10.91)
Sex, n (%)	
Female	7 (38.9)
Male	11 (61.1)
Employment status, n (%)	
Student	0 (0)
Housewife	3 (16.7)
Working	13 (72.2)
Retired	2 (11.1)
Diet type, n (%)	
Vegetarian	10 (55.6)
Nonvegetarian	8 (44.4)
Body fat mass (kg), mean (SD)	35.39 (6.38)
Weight (kg), mean (SD)	82.06 (9.66)
Skeletal muscle mass (kg), mean (SD)	25.45 (4.16)
Waist circumference (cm), mean (SD)	105.84 (7.64)
HbA_1c^a^_ (%), mean (SD)	8.10 (0.41)
Fasting plasma glucose (mg/dL), mean (SD)	143.68 (28.54)
Postprandial glucose (mg/dL), mean (SD)	194.16 (28.90)
Total cholesterol (mg/dL), mean (SD)	138.61 (30.43)
Triglycerides (mg/dL), mean (SD)	174.95 (72.14)
HDL^b^ cholesterol (mg/dL), mean (SD)	41.16 (11.11)
LDL^c^ cholesterol (mg/dL), mean (SD)	74.47 (25.07)
VLDL^d^ cholesterol (mg/dL), mean (SD)	45.80 (28.36)
TIR^e^ (%), mean (SD)	74.37 (12.14)
TBR^f^ (%), mean (SD)	3.47 (3.29)
TAR^g^ (%), mean (SD)	22.16 (13.43)

a HbA_1c_: hemoglobin A_1c_.

b HDL: high-density lipoprotein.

c LDL: low-density lipoprotein.

d VLDL: very low-density lipoprotein.

e TIR: time in range.

f TBR: time below range.

g TAR: time above range.

### Primary Outcomes

#### Glycemic Control

At 90 days, mean HbA_1c_ decreased by 0.59 percentage points (95% CI 0.00-1.18; *P*=.05), representing a relative reduction of 7.3%. Fasting plasma glucose declined by 10.94 mg/dL (95% CI −11.0 to 32.9; *P*=.32; the relative percentage change from baseline to 90 days was −7.6%) and postprandial glucose by 25.42 mg/dL (95% CI −1.5 to 52.3), with a trend toward statistical significance (*P*=.06; −13.1%). CGM metrics showed a 9.98% relative increase in TIR from 74.37% to 81.79% (*P*=.16), a 32.3% reduction in TAR from 22.16% to 15.00% (*P*=.20), and no significant change in TBR (*P*=.84; Table 2).

**Table 2.** Changes in glycemic and continuous glucose monitoring parameters from baseline to 90 days (N=18).

Parameter	Baseline, mean (SD)	90 days, mean (SD)	Absolute change (95% CI)	Percentage change	*P* value
HbA_1c^a^_ (%)	8.10 (0.41)	7.51 (1.17)	−0.59 (0.00 to −1.18)	−7.28	.05^b^
FPG^c^ (mg/dL)	143.68 (28.54)	132.74 (31.98)	−10.94 (−11.0 to 32.9)	−7.61	.32
PPG^d^ (mg/dL)	194.16 (28.90)	168.74 (42.34)	−25.42 (−1.5 to 52.3)	−13.09	.06
TIR^e^ (%)	74.37 (12.14)	81.79 (16.89)	+7.42 (−3.4 to 18.2)	+9.98	.16
TBR^f^ (%)	3.47 (3.29)	3.74 (3.38)	+0.27 (−2.6 to 3.1)	+7.78	.84
TAR^g^ (%)	22.16 (13.43)	15.00 (17.72)	−7.16 (−4.0 to 18.3)	−32.31	.20

a HbA_1C_: hemoglobin A_1c_.

b Statistically significant.

c FPG: fasting plasma glucose.

d PPG: postprandial glucose.

e TIR: time in range.

f TBR: time below range.

g TAR: time above range.

#### Anthropometric Measures

Anthropometric outcomes indicated small reductions in adiposity while maintaining lean muscle mass. Body fat mass fell by 1.13 kg (−3.19%), waist circumference by 1.56 cm (−1.47%), and weight by 0.67 kg (−0.82%). Skeletal muscle mass increased marginally (+0.33 kg; +1.3%). None of these changes were statistically significant (Table 3).

**Table 3.** Changes in anthropometric measures from baseline to 90 days (N=18).

Parameter	Baseline, mean (SD)	90 days, mean (SD)	Absolute change (95% CI)	Percentage change	*P* value
Body fat mass (kg)	35.39 (6.38)	34.26 (7.82)	−1.13 (−4.1 to 6.3)	−3.19	.66
Skeletal muscle mass (kg)	25.45 (4.16)	25.78 (5.06)	+0.33 (−3.2 to 2.5)	+1.30	.84
Weight (kg)	82.06 (9.66)	81.39 (13.21)	−0.67 (−7.5 to 6.2)	−0.82	.88
Waist circumference (cm)	105.84 (7.64)	104.28 (9.95)	−1.56 (−4.6 to 7.7)	−1.47	.62

#### Lipid Profile

Lipid parameters showed modest but statistically substantial changes over 90 days. Low-density lipoprotein cholesterol decreased by 8.38 mg/dL (−11.25%), total cholesterol by 10.14 mg/dL (−7.32%), and very low-density lipoprotein by 9.06 mg/dL (−19.78%). High-density lipoprotein cholesterol remained stable, and triglycerides increased slightly by 8.73 mg/dL (+4.99%; Table 4).

**Table 4.** Changes in lipid profile from baseline to 90 days (N=18).

Parameter	Baseline, mean (SD)	90 days, mean (SD)	Absolute change (95% CI)	Percentage change	*P* value
Total cholesterol (mg/dL)	138.61 (30.43)	128.47 (25.91)	−10.14 (−12.0 to 32.3)	−7.32	.36
Triglycerides (mg/dL)	174.95 (72.14)	183.68 (59.27)	+8.73 (−41.2 to 23.7)	+4.99	.72
HDL^a^ cholesterol (mg/dL)	41.16 (11.11)	41.21 (9.26)	+0.05 (−4.9 to 4.8)	+0.12	.99
LDL^b^ cholesterol (mg/dL)	74.47 (25.07)	66.09 (23.44)	−8.38 (−10.7 to 27.5)	−11.25	.38
VLDL^c^ cholesterol (mg/dL)	45.80 (28.36)	36.74 (20.88)	−9.06 (−12.0 to 30.1)	−19.78	.48

a HDL: high-density lipoprotein.

b LDL: low-density lipoprotein.

c VLDL: very low-density lipoprotein.

### Secondary Outcomes

Over the 90-day program, participants reported improvements across multiple QoL domains. Sleep quality increased by 18.9 points (95% CI 61.11-80.00; *P*=.04), motivation by 13.06 points (95% CI 59.44-72.50; *P*=.06), stress management by 12.78 points (95% CI 65.55-78.33; *P*=.08), and mood by 10.55 points (95% CI 66.11-76.66; *P*=.12). A composite CBT score, encompassing 6 domains (mood, motivation, time management, stress management, sleep, and awareness), improved in all participants, with improvements ranging from 4.3% to 80%. Half of the cohort achieved a ≥20% increase, indicating a broad enhancement in psychosocial well-being linked to personalized lifestyle coaching and CGM-driven feedback (Table 5).

Improvement in HbA_1c_ was observed in 15 (83.3%) of the 18 participants. Among those who increased their intake of nutrient-rich foods, 92.9% (n=17) achieved an improvement in HbA_1c_ compared with 50% (n=9) of participants who did not make such dietary changes. All participants who reduced their consumption of EDNP foods demonstrated improvement in HbA_1c_, whereas none of the participants who maintained or increased EDNP intake showed any improvement. Similarly, 90% (n=16) of participants engaging in regular weekly exercise achieved HbA_1c_ improvement compared with 75% (n=13) of those who were not exercising regularly (Table 6).

At 3 months, HbA_1c_ reduction was the most frequently observed HEOR outcome, occurring in 75% (13/18) of participants, including 2 participants who were receiving insulin therapy at baseline. However, nearly 25% (4/18) of participants were nonresponders and did not show improvement in glycemic parameters. In comparison, follow-up interval extension and weight reduction were observed in 25% (4/18) and 61% (11/18) of participants, respectively. Additionally, both participants who were receiving insulin therapy at baseline showed a reduction in insulin doses at the end of the study.

**Table 5.** Changes in quality of life scores from baseline to 90 days.

Domain	Baseline, mean (SD)	90 days, mean (SD)	Absolute change	Percentage change	*P* value
Sleep quality	61.11 (26.1)	78.46 (10.7)	+18.89	+30.9	.04
Motivation	59.44 (21.0)	72.86 (10.7)	+13.06	+22.0	.06
Stress management	65.55 (24.8)	79.23 (10.4)	+12.78	+19.5	.08
Mood	66.11 (22.7)	76.92 (12.5)	+10.55	+15.9	.12

**Table 6.** Relationship between behavioral factors and hemoglobin A_1c_ (HbA_1c_) improvement (N=18).

Behavioral factor	HbA_1c_ improved, n/N (%)	HbA_1c_ not improved, n/N (%)	Total, n/N (%)
Increased NRF^a^ intake	13/14 (92.9)	1/14 (7.1)	14/18 (77.8)
No increase in NRF intake	2/4 (50)	2/4 (50)	4/18 (22.2)
Reduced EDNP^b^ intake	15/15 (100)	0/15 (0)	15/18 (83.3)
No reduction in EDNP intake	0/3 (0)	3/3 (100)	3/18 (16.7)
Regular weekly exercise	9/10 (90)	1/10 (10)	10/18 (55.6)
Not regular weekly exercise	6/8 (75)	2/8 (25)	8/18 (44.4)

a NRF: nutrient-rich foods.

b EDNP: energy-dense, nutrient-poor.

## Discussion

### Principal Findings

The GLIDE-C program is the first to evaluate a CGM-enabled, multidisciplinary digital therapeutic integrating nutrition, physiotherapy, and CBT with simultaneous assessment of clinical, behavioral, and QoL outcomes in a real-world Indian T2DM cohort, a population that carries a disproportionate share of global diabetes burden that remains underrepresented in digital therapeutics research.

We observed a 0.59-point reduction in HbA_1c_, an almost 10% rise in TIR, and a 32% fall in TAR, with 83% (15/18) of participants improving their glycemic control. These metabolic gains were accompanied by improvements in mood, motivation, sleep, stress, and anthropometric parameters, without loss of muscle mass.

CGM transforms glucose data from episodic snapshots into dynamic profiles, allowing users to see the glycemic impact of specific foods, physical activities, and stressors. This immediate feedback supports “closed-loop” learning, reinforcing beneficial behaviors and discouraging detrimental ones [12]. In contrast, traditional fingerstick self-monitoring provides limited context and often fails to motivate sustained change. CGM provides visual feedback that reduces the cognitive burden associated with interpreting the effects of dietary choices and physical activity, thereby lowering behavioral barriers to effective self-management [4,14]. Positive trends in the app serve as positive reinforcements and create an aspiration for self-comparison [7,12].

Reviews note that the high upfront cost of CGM is often offset by fewer complications and hospitalizations, as it reduces both hyperglycemia and hypoglycemia [14]. Randomized and real-world studies show that CGM reduces HbA_1c_ by 0.25% to 3% and increases TIR by 15% to 34%, while also reducing hypoglycemia and improving treatment satisfaction [14]. In the BlueStar real-time CGM program, adults with poor baseline control increased TIR by 15 percentage points and reduced hyperglycemia by 14 percentage points over 3 months, despite limited weekly engagement; participants attributed their success to “teachable moments” from real-time glucose feedback and artificial intelligence–driven coaching [12]. A 7.42-point rise in TIR in this study is comparable, given the shorter duration and relatively good baseline control. Studies combining CGM with digital coaching report similar or greater effects: January AI and January V2 (artificial intelligence–supported CGM mobile apps) increased TIR by 10% and achieved modest weight loss in cohorts tracked for 14 to 28 days [15,16]. The digital twin program, which leverages machine learning and IoT sensors to deliver year-long personalized nutrition and activity plans, achieved a 1.8% HbA_1c_ reduction, weight loss of 4.8 kg, and a reduction in antidiabetic medication use from 1.9 to 0.5 drugs per person; nearly 89% of participants attained HbA_1c_ <7% [17]. Digital applications that focus on a single behavioral domain yield a more limited glycemic effect. In a 12-week randomized trial of a smartphone personal health record app with motivational SMS text messages, HbA_1c_ reduced from 6.9% to 6.7% in the intervention arm but remained similar in the control arm, and the primary outcome, step count, did not differ [18]. A 6-month trial of the Emperra GmbH E-Health Technologies digital application (largely for insulin-treated patients) reported a 0.48% reduction in HbA_1c_ vs a 0.28% difference relative to standard care [19]. More recently, the Vitadio mobile health app, which combines educational content, self-monitoring, and virtual coaching, was associated with a greater reduction in HbA1c than standard care (−0.8% vs −0.3%), comparable to the findings of this study, as well as significant reductions in weight and BMI and improvements in self-care practices [20].

Individuals who increase consumption of nutrient-rich foods typically achieve greater HbA_1c_ improvement, with average reductions of about 0.3% to 0.4%, compared with those who do not, while those who reduce EDNP foods consistently show improvements of around 0.5% [21,22]. These findings align with digital nutrition interventions such as Vitadio and in our GLIDE-C study, where significant improvements in diet quality drove HbA_1c_ reduction and weight loss [20]. Regular exercise is also associated with HbA_1c_ improvement; this mirrors data from physical activity apps, where participants with low baseline activity achieved greater step count and HbA_1c_ improvements [18].

The individual plan, including CBT for all participants, supports and fosters autonomous motivation, which may empower patients in the long term [8]. These findings suggest that sustained engagement with data-driven programs can yield durable metabolic benefits through behavior modification [16,17], leading to improvements in health-related QoL and, in some cases, medication de-escalation as clinical outcomes improve, as observed in this study [9,13]. Additionally, in this study, reductions in HbA_1c_ and body weight were associated with reduction in insulin doses and longer intervals between follow-up visits, collectively reflecting an overall improvement in HEOR outcomes.

This study implements the synergistic effects of diet quality, exercise, CBT, and IoT devices through a single digital platform unifying all the interventions from available literature. South Asian individuals characterized by insulin resistance at lower BMI thresholds and earlier onset of T2DM may respond differently to lifestyle-based digital interventions compared with Western populations [2,3,11]. The findings of this study therefore carry specific translational relevance for health systems in India and the broader South Asian region, where scalable, low-infrastructure digital solutions are urgently needed [1,10]. This real-world observational cohort study provides preliminary evidence for a CGM-integrated digital therapeutic application. The single-arm design and the current sample size are consistent with published feasibility studies and sufficiently detect clinically meaningful improvements across glycemic and behavioral domains [7,8,10,11,15]. However, the absence of a randomized control group precludes causal inference and may reflect insufficient statistical power for secondary end points, while regression to the mean cannot be excluded. The time frame, though standard in digital therapeutics trials, captured early program effects; longer follow-up in future studies will enable us to characterize the sustainability of outcomes.

### Conclusions

The novel GLIDE-C program, integrating CGM with personalized nutrition, physiotherapy, and CBT, achieved meaningful improvements in glycemic control, QoL, and health behaviors in adults with T2DM. Real-time data review and tailored coaching fostered dietary and activity changes that were strongly associated with reductions in HbA_1c_. These findings highlight the potential of holistic, app-based digital therapeutics to complement conventional diabetes care and support sustainable lifestyle modification in real-world settings.

## Abbreviations

CGM: continuous glucose monitoring
HbA_1c_: hemoglobin A_1c_
HEOR: health economics and outcomes research
IoT: Internet of Things
QoL: quality of life
SMBG: self-monitoring of blood glucose
T2DM: type 2 diabetes mellitus
TAR: time above range
TBR: time below range
TIR: time in range
